# Masticatory sensory-motor changes after an experimental chewing test influenced by pain catastrophizing and neck-pain-related disability in patients with headache attributed to temporomandibular disorders

**DOI:** 10.1186/s10194-015-0500-1

**Published:** 2015-03-05

**Authors:** Roy La Touche, Alba Paris-Alemany, Alfonso Gil-Martínez, Joaquín Pardo-Montero, Santiago Angulo-Díaz-Parreño, Josué Fernández-Carnero

**Affiliations:** Department of Physiotherapy, Faculty of Health Science, The Center for Advanced Studies University La Salle, Universidad Autónoma de Madrid, Aravaca, Madrid, Spain; Motion in Brains Research Group, The Center for Advanced Studies University La Salle, Universidad Autónoma de Madird, Aravaca, Madrid, Spain; Institute of Neuroscience and Craniofacial Pain (INDCRAN), Madrid, Spain; Hospital La Paz Institute for Health Research, IdiPAZ, Madrid, Spain; Faculty of Medicine, Universidad San Pablo CEU, Madrid, Spain; Department of Physical Therapy, Occupational Therapy, Rehabilitation and Physical Medicine, Universidad Rey Juan Carlos, Alcorcón, Madrid, Spain

**Keywords:** Temporomandibular disorders, Headache, Neck pain, Pain catastrophizing, Disability

## Abstract

**Background:**

Recent research has shown a relationship of craniomandibular disability with neck-pain-related disability has been shown. However, there is still insufficient information demonstrating the influence of neck pain and disability in the sensory-motor activity in patients with headache attributed to temporomandibular disorders (TMD). The purpose of this study was to investigate the influence of neck-pain-related disability on masticatory sensory-motor variables.

**Methods:**

An experimental case–control study investigated 83 patients with headache attributed to TMD and 39 healthy controls. Patients were grouped according to their scores on the neck disability index (NDI) (mild and moderate neck disability). Initial assessment included the pain catastrophizing scale and the Headache Impact Test-6. The protocol consisted of baseline measurements of pressure pain thresholds (PPT) and pain-free maximum mouth opening (MMO). Individuals were asked to perform the provocation chewing test, and measurements were taken immediately after and 24 hours later. During the test, patients were assessed for subjective feelings of fatigue (VAFS) and pain intensity.

**Results:**

VAFS was higher at 6 minutes (mean 51.7; 95% CI: 50.15-53.26) and 24 hours after (21.08; 95% CI: 18.6-23.5) for the group showing moderate neck disability compared with the mild neck disability group (6 minutes, 44.16; 95% CI 42.65-45.67/ 24 hours after, 14.3; 95% CI: 11.9-16.7) and the control group. The analysis shows a decrease in the pain-free MMO only in the group of moderate disability 24 hours after the test. PPTs of the trigeminal region decreased immediately in all groups, whereas at 24 hours, a decrease was observed in only the groups of patients. PPTs of the cervical region decreased in only the group with moderate neck disability 24 hours after the test. The strongest negative correlation was found between pain-free MMO immediately after the test and NDI in both the mild (r = −0.49) and moderate (r = −0.54) neck disability groups. VAFS was predicted by catastrophizing, explaining 17% of the variance in the moderate neck disability group and 12% in the mild neck disability group.

**Conclusion:**

Neck-pain-related disability and pain catastrophizing have an influence on the sensory-motor variables evaluated in patients with headache attributed to TMD.

## Background

Recent research has shown a strong relationship of craniomandibular pain and disability with neck-pain-related disability [1,2]. However, there is still insufficient information demonstrating the influence of neck pain and disability in the sensory-motor activity in patients with headache attributed to temporomandibular disorders (TMD). Our primary hypothesis is that neck disability is a factor that influences masticatory sensory-motor activity in patients with headache attributed to TMD.

Headache attributed to TMD is classified as a secondary headache caused by a disorder that affects the temporomandibular region [3]. The pain may be unilateral or bilateral and is represented in the masseter and temporal regions of the face [3]. An important criterion for clinical diagnosis is that headache is caused or is aggravated by provocative manoeuvres (such as palpatory pressure on the TMJ and masticatory muscles) or mandibular active or passive movements [3,4]. Recently, it was found that the diagnostic criteria that have greater sensitivity and specificity for this type of headache are: 1) the provocation of pain by palpation of the temporalis muscle or jaw movements and 2) changes in pain with the movements of the jaw in the function or parafunction [4,5]. From a clinical point of view, it is important to identify changes in motor behaviour that may be present in patients suffering TMD, especially knowing that a percentage of these patients develop painful chewing [6,7], difficulty performing jaw movements [8], and masticatory fatigue [9,10].

The relationship between masticatory muscle pain and disordered jaw motor behaviour has widely been studied during the last few decades. For example, see the review by Svensson and Graven-Nielsen [11]. Pain may influence the characteristics of the masticatory sensory-motor system [12]. Kurita et al. found a positive correlation between chewing ability, TMJ pain, and reduced mouth opening [13]. According to some researchers, fatigue and fatigue-related symptoms are reported significantly more often by chronic TMD patients than by healthy volunteers [14]. In addition, a recent study on patients with chronic orofacial pain demonstrated that fatigue is mediated by psychosocial factors [15]. For example, Brandini et al. found a positive association in TMD patients between mandibular kinematic variables and psychological factors such as stress and depression [16].

Research or assessments based on a biobehavioural approach may offer a better alternative for identifying patients with chronic TMD [17]. The biobehavioural approach to the assessment and treatment of chronic pain is widely accepted [18]. A key point to note about patients with headache attributed to TMD is the association between emotional functioning and increased frequency of headache [19]. This and previous findings lead us to propose that the assessment of psychological factors be integrated with pain and disability associated with masticatory sensory-motor variables in this research. A significant amount of scientific evidence has shown the influence of pain catastrophizing on several variables related to TMD [20-25]. This leads to the hypothesis that pain catastrophizing has an association with or is a predictor of some of the masticatory sensory-motor variables.

The primary objective of this research is to investigate the influence that pain and disability of the neck may have on masticatory sensory-motor variables in patients with headache attributed to TMD. As a secondary objective, we propose identifying whether the psychological or disability variables have any association with the studied sensory-motor variables.

## Methods

### Study design

This was an experimental case–control study. The assessor of sensory-motor measurements was blinded. One researcher administered the participant appointments and questionnaires and instructed the participants not to say anything that could reveal their pain, disability trait, or state. The reporting of the study follows the “Strengthening the Reporting of Observational Studies in Epidemiology” (STROBE statement) [26]. After receiving detailed information about the experiment, the volunteers gave their written informed consent. All of the procedures were planned under the ethical norms of the Helsinki Declaration and were approved by the local ethics committee.

### Participants

A consecutive convenience sample of 83 patients with chronic headache attributed to TMD and 39 healthy controls were recruited. The sample was recruited from outpatients of a public health centre (Madrid, Spain) and two private clinics specializing in craniofacial pain and TMD (Madrid, Spain). Patients were selected if they met all of the following criteria: 1) Headache and facial pain attributed to TMD with diagnosis according to the guidelines of the International Classification of Headache Disorders [3]; 2) TMD diagnosis based on the Research Diagnostic Criteria for TMD [27,28] to classify patients with painful TMD (myofascial pain, TMJ arthralgia, and TMJ osteoarthritis); 3) history of pain symptoms in at least the 6 months prior to the study; 4) pain in the jaw, temples, face, neck, pre-auricular area, or in the ear during rest or function; 5) neck pain and disability quantified according to the neck disability index (NDI) [29]; and 6) at least 18 years of age.

There were 83 patients categorized into two groups according to their scores on the NDI [29]: 1) the mild neck disability group (NDI 5–14) and 2) the moderate neck disability group (NDI 15–24). The criteria for exclusion were: 1) a history of traumatic injuries (e.g., contusion, fracture, or whiplash injury); 2) presence of fibromyalgia or other chronic pain disorder; 3) neuropathic pain (e.g., trigeminal neuralgia); 4) unilateral neck pain; 5) cervical spine surgery, and 6) clinical diagnosis of cervical radiculopathy or myelopathy.

Healthy controls were recruited from our academic university campus and the local community through flyers, posters, and social media. Healthy participants were examined and included in the study only if they had no history of craniofacial pain, headache, or neck pain, and had been free of any other painful disorders for the six months prior to the experiment. All subjects had complete dentition, did not use any medication, had no dental pathology, and did not chew gum regularly. Subjects who reported oral parafunctions (i.e., tooth grinding, tooth clenching) were excluded.

### Experimental protocol

After consenting to the study, recruited patients were given a battery of questionnaires to complete on the first day of the experiment. These included various self-reports for sociodemographic, psychological, and pain-related variables, including the visual analogue scale (VAS) for pain intensity, the validated Spanish versions of the pain catastrophizing scale (PCS), the NDI, and the impact associated with headache was assessed using the Headache Impact Test-6 (HIT-6). The experimental protocol consisted of baseline measurements, a provocation chewing test, and data collection immediately after the provocation chewing test and 24 hours later. Participants underwent standardized measurement of pressure pain thresholds (PPT) for mechanical pain sensitivity at the trigeminal and cervical region and of pain-free maximum mouth opening (MMO). The PPT and MMO measures have been employed in previous studies [30]. During the performance of the provocation chewing test, data were collected regarding the subjective feelings of fatigue and pain intensity every minute, immediately after the test, and 24 hours later.

### Provocation chewing test

The provocation chewing test consisted of 6 minutes of unilateral chewing of eight grams of hard gum, a protocol that was modified from Karibe et al. [31]. Chewing gum was employed to elicit pain and muscle fatigue. The participants performed the test in the sitting position, which was attained by instructing the patient to sit in a comfortable upright position with the thoracic spine in contact with the back of the chair, but without contact of the craniocervical region with the seat. The feet were positioned flat on the floor with knees and hips at 90 degrees and arms resting freely alongside.

Tests were carried out using the right side for chewing exclusively. A metronome was set at 80 beats per minute to indicate chewing rate, as documented in a previous study [32]. The participants were instructed to chew gum initially for 60 seconds to soften its initial hardness, then after 70 seconds of rest, the signal was given to start the test.

### Questionnaires

The Spanish version of the PCS assesses the degree of pain catastrophizing [33,34]. The PCS has 13 items and a 3-factor structure: rumination, magnification, and helplessness. The theoretical range is between 0 and 52, with lower scores indicating less catastrophizing. The PCS has demonstrated acceptable psychometric properties [34].

The Spanish version of the NDI measures perceived neck disability [29,35] is a questionnaire that consists of 10 items, with 6 possible answers that represent 6 levels of functional capacity ranging from 0 (no disability) to 5 (complete disability) points. The sum of all of the points obtained from each of the items gives the level of disability, with higher scores indicating greater perceived disability. The NDI has demonstrated acceptable psychometric properties [35].

The Spanish version of the HIT-6 [36,37] consists of a six-item questionnaire that measures the severity and impact of headache on the patient’s life. The results of HIT-6 are stratified into four grade-based classes: little or no impact (HIT-6 score: 36–49), moderate impact (HIT-6 score: 50–55), substantial impact (HIT-6 score: 56–59), and severe impact (HIT-6 score: 60–78) [36]. The HIT-6 has demonstrated acceptable psychometric properties [38].

### Pain intensity

Pain intensity was measured with the VAS. The VAS consists of a 100-mm line on which the left side represents “no pain” and the right side represents “the worst pain imaginable”. The patients placed a mark to represent their pain intensity [39]. VAS was used to quantify two different situations:

- Habitual and spontaneously perceived pain intensity.
- Pain intensity perceived at different times during the course of the chewing provocation test and at 24 h after completion.

### Subjective perception of fatigue

The visual analogue fatigue scale (VAFS) was used to quantify fatigue at different times during the course of the chewing provocation test and at 24 h after completion. The VAFS consists of a 100-mm vertical line on which the bottom represents “no fatigue” (0 mm), and the top represents “maximum fatigue” (100 mm) [40]. The researcher registered the mark in mm.

### Pressure pain thresholds

A digital algometer (FDX 25, Wagner Instruments, Greenwich, CT, USA) comprising a rubber head (1 cm^2^) attached to a pressure gauge was used to measure PPTs. Force was measured in kilograms (kg), and thresholds were expressed in kg/cm^2^. The protocol used was a sequence of 3 measurements with an interval of 30 seconds between each of the measurements. The average of the 3 measurements was calculated to obtain a single value for each of the measured points in each of the assessments. PPTs were assessed at one point in the masseter muscle (2.5 cm anterior to the tragus and 1.5 cm inferior to the zygomatic arch), one point in the temporalis muscle (anterior fibres of the muscle; 3 cm superior to the zygomatic arch in the middle point between the end of the eye and the anterior part of the helix of the ear), in the suboccipital muscles (2 cm inferior and lateral to the external occipital protuberance), and in the upper trapezius muscle (2.5 cm above the superior medial angle of the scapula). The device was applied perpendicular to the skin. The patients were asked to raise their hands at the moment the pressure started to change to a pain sensation, at which point the assessor stopped applying pressure. Compression pressure was gradually increased at a rate of approximately 1 kg/s. This algometric method has high intra-rater reliability (ICC = 0.94-0.97) for measuring PPT [41].

### Pain-free MMO

MMO was measured with the patients in a supine position. The patients were asked to open their mouths as widely as they could without pain. The distance between the superior incisor and the opposite inferior incisor was measured in mm with a craniomandibular scale (CMD scale. Pat. No. ES 1075174 U, INDCRAN: 2011. INDCRAN, Madrid, Spain). The inter-rater reliability of this procedure has been found to be high (ICC = 0.95 – 0.96) [42].

### Sample size

The sample size was estimated with the program G*Power 3.1.7 for Windows (G*Power© from University of Dusseldorf, Germany) [43]. The sample size calculation was considered as a power calculation to detect between-group differences in the primary outcome measures (fatigue and pain intensity). We considered 3 groups and 7 measurements for primary outcomes to obtain 80% statistical power (1-β error probability) with an α error level probability of 0.05 using analysis of variance (ANOVA) of repeated measures, within-between interaction, and a medium effect size of 0.3. This generated a sample size of 31 participants per group. Allowing a dropout rate of 20% and aiming to increase the statistical power of the results, we planned to recruit a minimum of 112 participants to provide sufficient power to detect significant group differences.

### Statistical analysis

The Statistical Package for Social Sciences (SPSS 21, SPSS Inc., Chicago, IL USA) software was used for statistical analysis. The independent t-test and one-way ANOVA were used for analysis of the self-report psychological and pain-related variables (NDI, PCS and HIT-6), as well as pain duration and the subjects’ sociodemographic data (age, weight, height). The baseline data were compared for the three groups. Results are presented as the mean, standard deviation (±SD), range, and 95% confidence interval (CI).

For primary outcome variables (fatigue and pain intensity), we performed a 3-way repeated-measures ANOVA, including within-between interaction factors. The factors analysed were group (moderate neck disability group, mild neck disability group, and healthy group), sex (female and male), and time (measurement per minute during the test and after 24 hours). The hypothesis of interest was the group *vs*. time interaction.

The 2-way repeated-measures models of ANOVA were used to test the effect of the task on the outcome secondary variables (i.e., PPTs and pain-free MMO). The factors analysed were group and time (baseline, immediately after, and after 24 hours). The interactions of group *vs.* time were also analysed. In the repeated-measures ANOVAs, when the assumption of sphericity was violated (as assessed using the Mauchly sphericity test), the number of degrees of freedom against which the F-ratio was tested was corrected by the value of the Greenhouse–Geisser adjustment. Post hoc analysis with Bonferroni corrections was performed in the case of significant ANOVA findings for multiple comparisons between variables. Effect-sizes (Cohen’s *d*) were calculated for outcome secondary variables. According to Cohen’s method, the magnitude of the effect was classified as small (0.20 to 0.49), medium (0.50 to 0.79), or large (≥0.8) [44].

The relationship between pain-related measures after completion of the chewing provocation test and self-reports for pain-related and psychological measures were examined using Pearson correlation coefficients. Multiple linear regression analysis was performed to estimate the strength of the associations between the results of VAS [model 1], VAFS [model 2], and pain-free MMO [model 3] (criterion variables) after 24 hours following completion of the provocation chewing test. NDI, PCS, HIT-6, and VAS were used as predictor variables. Variance inflation factors (VIFs) were calculated to determine whether there were any multi-collinearity issues in any of the three models.

The strength of association was examined using regression coefficients (β), P values, and adjusted R^2^. Standardized beta coefficients were reported for each predictor variable included in the final reduced models to allow for direct comparison between the predictor variables in the regression model and the criterion variable being studied. For regression analysis, the rule of 10 cases per variable was applied in order to obtain reasonably stable estimates of the regression coefficients [45]. The significance level for all tests was set to *P* < 0.05.

## Results

Baseline characteristics of sociodemographic, psychological, and pain-related variables of the sample are summarized in Table 1. Finally, the total study sample consisted of 122 participants (77 females and 45 males). Table 1 shows no statistically significant differences among the three groups in relation to sociodemographic variables. There were no differences in the duration of pain and perceived pain intensity on a regular or spontaneous basis in specific groups of patients, but differences were observed in NDI, PCS, and Hit-6 (p < 0.05). The different diagnoses for TMD of the included patients were as follows: 28 patients (33.7%) were diagnosed with myofascial pain, 8 patients (9.6%) with arthralgia, 13 patients (15.6%) with osteoarthritis, and 34 patients (40.9%) with a combined diagnosis (myofascial pain with arthralgia or osteoarthritis).

**Table 1:** **Summary of demographic, pain and psychological variables**

	**Moderate neck disability (N = 41)**		**Mild neck disability (N = 42)**		**Healthy (N = 39)**		**t/F**	**P value**
**Variables**	**Mean ± SD**	**Range**	**Mean ± SD**	**Range**	**Mean ± SD**	**Range**		
Sex (female/male)	26/15	-	25/17	-	26/13	-	-	-
Weight (kg)	69.56 ± 12.47	51-103	67.76 ± 14.03	50-97	64.84 ± 10.2	48-90	1.4	0.23
Heigth (cm)	167.56 ± 12.47	152-183	165.54 ± 12.09	150-185	169.97 ± 8.51	156-189	1.98	0.14
Age (years)	44.31 ± 10.9	22-59	40.95 ± 12.89	19-60	40.61 ± 10.01	30-65	1.3	0.27
Pain duration (months)	19.73 ± 12.66	6-60	22.19 ± 13.36	6-48	-	-	−0.8	0.39
NDI (points)	17.58 ± 2.69	15-24	11.42 ± 2.48	7-14	-	-	10.8	0.01
PCS (points)	17.09 ± 3.75	7-23	15.8 ± 4.02	7-22	5.46 ± 1.75	2-9	143	0.01
HIT-6 (points)	55.31 ± 4.9	49-65	53.16 ± 4.74	43-59	-	-	2	0.04
VAS (mm)	40.75 ± 9.17	21-58	37.04 ± 9.16	19-54	-	-	1.8	0.06

*Abbreviations*: *NDI* neck disability index, *PCS* pain catastrophizing scale, *HIT-6* headache impact test-6, *VAS* Visual Analog Scale, *SD* standard deviation.

In the group of healthy participants, there were no withdrawals during the provocation chewing test. In the group of patients with moderate neck disability, nine participants (21.9%) withdrew between minutes 5 and 6 of the test, as did six participants in the group of patients with mild neck disability (14.2%). All of the participants were evaluated 24 hours after the test.

### Gender differences in response to provocation chewing test

The interaction of group *vs.* sex showed significant differences in VAS (F = 10.86; P < 0.001), VAFS (F = 4.06; P = 0.02), and PPTs of the trapezius muscle (F = 3.96; P = 0.022). *Post hoc* analysis showed higher values of VAS and VAFS in women compared to men for the three groups (P < 0.05). PPTs in the trapezius muscle values were lower in women than in men (P < 0.05) for the two groups of patients, but in the control group, there was no difference in this value. No differences (group *vs.* sex interaction) were observed for the other variables.

### Pain and fatigue

**Figure 1:**
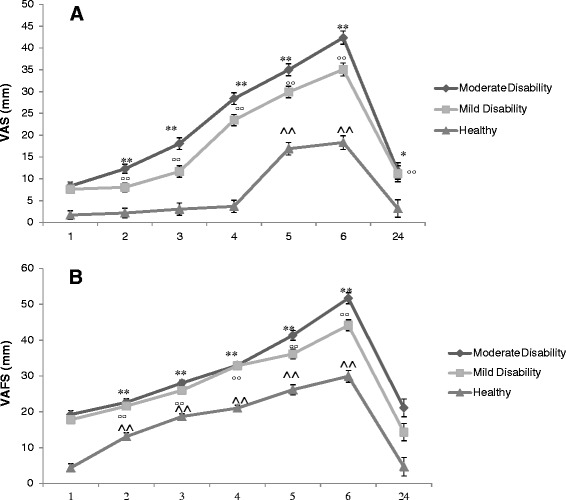
**Data represent mean value and error bars with 95% confidence intervals of the mean of the pain intensity score (A), and the visual analogue fatigue scale scores (B).** Recorded during the 6 min and 24 hours after provocation chewing test. Level of significance (multiple comparisons for each group): Moderate disability, *P < 0.05; **P < 0.01; Mild disability, °P < 0.05; °°P < 0.01; and Healthy, ^P < 0.05; ^^P < 0.01.

**Figure 2:**
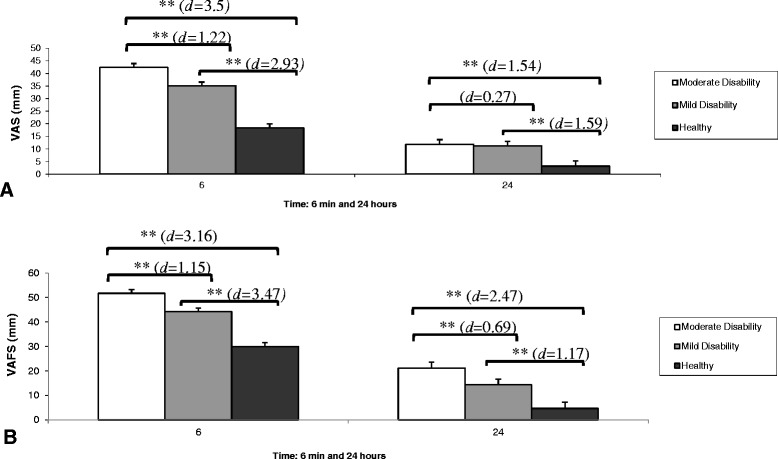
**Comparison between groups of the pain intensity (A) and perceived fatigue (B) immediately (6 min) and 24 hours after the provocation chewing test.** Data represent mean value, error bars with 95% confidence intervals of the mean and effect size (*d*). Level of significance: *P < 0.05; **P < 0.01.

The ANOVA revealed a significant group *vs.* time interaction (F = 35.77; P < 0.001), and significant differences for the group factor (F = 416.65; P < 0.001) regarding the VAS results during the provocation chewing test. VAS behaviour during the tests can be seen in Figure 1A. *Post hoc* analysis revealed higher values on the VAS during the provocation chewing test for the moderate neck disability group compared to the mild neck disability group and the control group. The results obtained 24 hours after the test showed no differences between the groups of patients, but there were differences with the control group (Figure 2A).

For fatigue perceived during tests, the ANOVA showed a significant effect for group *vs.* time interaction (F = 13.05; P < 0.001) and for the group factor (F = 371.12; P < 0.001). VAFS behaviour during the tests can be seen in Figure 1B. VAFS values were higher at 6 minutes and 24 hours after the test in the group of moderate neck disability compared with the other two groups. The *post hoc* analysis shows the differences between the three groups (Figure 2B). Table 2 shows the percentages of patients that achieved a significant change in pain intensity and fatigue perceived after 6 minutes of intense chewing.

**Table 2:** **Changes in pain intensity and perception of fatigue immediate after experimental chewing test**

	**Pain intensity**			**Perception of fatigue**		
	**% of participants (N)**			**% of participants (N)**		
	**Moderate neck disability**	**Mild neck disability**	**Healthy**	**Moderate neck disability**	**Mild neck disability**	**Healthy**
**Increase**	100% (41)	92.8% (39)	60.5% (23)	100% (41)	97.6% (41)	84.2% (32)
**No change**	0% (0)	7.14% (3)	39.5% (15)	0% (0)	2.3% (1)	15.8% (6)

### Pain-free MMO

Regarding the pain-free MMO, ANOVA revealed a significant effect for group *vs.* time interaction (F = 2.75; P = 0.02) and for the group factor (F = 65.74; P < 0.001). The *post hoc* analysis shows a decrease in the pain-free MMO immediately after the tests for the three groups, but for 24 hours after the test, this decrease was observed in only the group of moderate disability (Table 3).

**Table 3:** **Descriptive data and multiple comparisons of the assessed variables**

		**Mean ± SD**			**Mean difference (95% CI) effect size** ***(d)***
	**Group**	**Baseline**	**Immediately after**	**After 24 hours**	**a) Base vs. immediately**
					**b) Base vs. 24 h**
**MMO (mm)**	**Moderate Neck Disability**	42.43 ± 2.75	40.65 ± 2.01	41.85 ± 2.19	a) 1.89 (1.39 to 2.39)**; *d* = 0.74
					b) 0.6 (0.02 to 1.17)*; *d* = 0.26
	**Mild Neck Disability**	43.61 ± 2.87	42 ± 2.18	43.26 ± 2.68	a) 1.56 (1.09 to 2.07)**; *d* = 0.63
					b) 0.36 (−0.01 to 0.75); *d* = 0.12
	**Healthy**	50 ± 4.46	49.05 ± 3.95	49.87 ± 4.57	a) 0.76 (0.24 to 1.29)*; *d* = 0.22
					b) 0.09 (−0.32 to 0.51); *d* = 0.02
**PPT. Masseter**	**Moderate Neck Disability**	1.9 ± 0.21	1.02 ± 0.17	0.88 ± 0.2	a) 0.89 (0.79 to 0.99)** *d* = 4.66
					b) 1.03 (0.94 to 1.13)** *d* = 5.03
	**Mild Neck Disability**	2.01 ± 0.34	1.44 ± 0.28	1.57 ± 0.34	a) 0.57 (0.48 to 0.67)** *d* = 1.82
					b) 0.44 (0.35 to 0.53)** *d* = 1.29
	**Healthy**	2.85 ± 0.58	2.39 ± 0.52	2.7 ± 0.51	a) 0.45 (0.35 to 0.56)** *d* ***=*** 0.84
					b) 0.13 (0.03 to 0.23)* *d* = 0.27
**PPT. Temporalis**	**Moderate Neck Disability**	1.99 ± 0.19	1.55 ± 0.25	1.62 ± 0.23	a) 0.44 (0.39 to 0.49)** *d* = 2.06
					b) 0.37 (0.25 to 0.49)** *d* = 1.77
	**Mild Neck Disability**	2.12 ± 0.35	2.05 ± 0.37	2.04 ± 0.45	a) 0.07 (0.02 to 0.12)** *d* = 0.19
					b) 0.09 (−0.02 to 0.2) *d* = 0.20
	**Healthy**	3.31 ± 0.83	3.26 ± 0.82	3.27 ± 0.84	a) 0.04 (−0.001to 0.1) *d* = 0.06
					b) 0.06 (−0.05 to 0.19) *d* = 0.04
**PPT. Suboccipital**	**Moderate Neck Disability**	2.39 ± 0.44	1.65 ± 0.36	1.47 ± 0.32	a) 0.78 (0.7 to 0.85)** *d* = 1.86
					b) 0.95 (0.83 to 1.07)** *d* = 2.42
	**Mild Neck Disability**	2.14 ± 0.57	2.06 ± 0.55	2.22 ± 0.57	a) 0.07 (−0.00 to 0.15) *d* = 0.14
					b) -0.11 (−0.23 to 0.00)* *d = 0.14*
	**Healthy**	3.15 ± 0.56	3.09 ± 0.55	3.18 ± 0.59	a) 0.06 (−0.01 to 0.14) *d* = 0.1
					b) -0.01 (−0.13 to 0.11) *d* = 0.05
**PPT. Trapezius**	**Moderate Neck Disability**	2.62 ± 0.49	2.33 ± 0.47	2.49 ± 0.45	a) 0.28 (0.24 to 0.33)** *d* = 0.61
					b) 0.14 (0.08 to 0.2)** *d* = 0.27
	**Mild Neck Disability**	2.68 ± 0.62	2.62 ± 0.63	2.63 ± 0.58	a) 0.04 (0.00 to 0.09)* *d* = 0.09
					b) 0.04 (−0.01 to 0.1) *d* = 0.08
	**Healthy**	3.54 ± 1	3.51 ± 0.97	3.53 ± 0.94	a)0.01 (−0.03 to 0.06) *d* = 0.03
					b) -0.01 (−0.05 to 0.07) *d* = 0.01

**p < 0.01; *p < 0.05.

*Abbreviations*: *MMO* maximal mouth opening, *PPT* pressure pain threshold, *SD* standard deviation.

### Pressure pain thresholds

The PPTs for all points of the trigeminal and cervical region showed statistically significant differences by ANOVA in the group *vs.* time interaction and group factor (P < 0.001). According to the *post hoc* analysis of the PPT masseter muscle, the results showed a decrease in all groups for measurements both immediately and 24 hours after the test (P < 0.05); however, this decrease was greater in the group showing moderate neck disability (*d* > 0.8). Changes in temporalis muscle PPT were observed in both measures for the group of moderate neck disability (P < 0.001; *d* > 0.8). In the group of mild neck disability, changes were observed only immediately after the test (P = 0.002; *d* = 0.19). No changes were observed in the group of healthy subjects (P > 0.05).

For PPT in the cervical region (trapezius muscle and suboccipital muscles), the *post hoc* analysis shows a decrease of values measured immediately and 24 hours after the test (P < 0.001) for the group of moderate neck disability. This decrease in PPT can be considered large for the suboccipital region (*d* > 0.9) and small to medium for the trapezius muscle (*d* = 0.27 and 0.61). In the group with mild neck disability, changes were observed in only the trapezius muscle PPT measurement immediately after the test (P = 0.028; *d* = 0.09), and no statistically significant differences were observed in any of the PPT measurements in the cervical region in the group of healthy subjects (P > 0.05).

### Correlations analysis

Table 4 shows the results of correlation analysis examining the bivariate relationships among self-reports for pain-related and psychological measures and MMO, VAS, and VAFS measured immediately and 24 hours after the tests for the groups with moderate and mild neck disability. The strongest correlations were found in the analysis for the group with moderate neck disability, where the pain-free MMO immediately after the test was negatively associated with NDI (r = −0.54; P < 0.001). For the mild neck disability group, the greater correlation was between the MMO results after 24 hours and NDI, which had a negative association (r = −0.49; P < 0.001).

**Table 4:** **Pearson’s correlation coefficient between the different variables analyzed in the study**

**Groups**		**VAS 6 min**	**VAS 24 h**	**VAFS 6 min**	**VAFS 24 h**	**MMO immediately after**	**MMO 24 h**
**Moderate neck disability**	NDI	0.49**	0.28	0.40**	0.07	−0.54**	−0.40**
**Mild neck disability**		0.02	0.37*	0.02	0.21	−0.48**	−0.49**
**Moderate neck disability**	PCS	0.10	0.24	0.17	0.44**	0.03	0.04
**Mild neck disability**		0.08	0.40**	0.01	0.38*	−0.17	−0.09
**Moderate neck disability**	HIT-6	0.41**	0.48**	0.27	0.07	−0.12	−0.31*
**Mild neck disability**		−0.11	−0.03	0.30	−0.04	−0.13	−0.12
**Moderate neck disability**	VAS	−0.08	0.39*	0.49**	0.16	−0.23	−0.25
**Mild neck disability**		−0.08	0.17	−0.17	0.11	−0.39*	−0.47**

**p < 0.01; *p < 0.05.

*Abbreviations*: *NDI* neck disability index, *PCS* pain catastrophizing scale, *HIT-6* headache impact test-6, *VAS* visual analog scale, *VAFS* visual analog fatigue scale, *MMO* maximal mouth opening.

### Multiple linear regression analysis

A linear regression analysis was performed to evaluate contributors to VAFS, VAS, and pain-free MMO after 24 hours regarding all of the self-report results for pain-related and psychological measures in the patient groups with moderate and mild neck disability. The results are presented in Table 5. In the first model, the criterion variable VAFS was predicted by pain catastrophizing (for both groups), explaining 17% and 12% of the variance, respectively. The following variables were not significant predictors: VAS (moderate neck disability, β = −0.001; P = 0.10, mild neck disability, β = −0.053; P = 0.72), HIT-6 (moderate neck disability, β = 0.004; P = −0.97, mild neck disability, β = −0.071; P = 0.63), and NDI (moderate neck disability, β = −0.082; P = 0.59, mild neck disability, β = −0.070; P = 0.67).

**Table 5:** **Multiple linear regression analysis**

**Criterion variable**	**Predictor variables**	**Regression coefficient (B)**	**Standardized coefficient (β)**	**Significance (p)**	**VIF**	**Adjusted R** ^**2**^
**Moderate neck disability**						
VAFS24	PCS	0.84	0.44	0.004	1.00	0.17
VAS24	HIT-6	0.93	0.48	0.001	1.00	0.22
MMO24	NDI	−0.35	−0.40	0.01	1.12	0.14
**Mild neck disability**						
VAFS24	PCS	0.98	0.38	0.013	1.00	0.12
VAS24	PCS	0.67	0.40	0.009	1.00	0.14
MMO24	NDI	−0.53	−0.49	0.001	1.00	0.21

*Abbreviations*: *NDI* neck disability index, *PCS* pain catastrophizing scale, *HIT-6* headache impact test-6, *VAS* visual analog scale, *VAFS* visual analog fatigue scale, *MMO* maximal mouth opening, *24* 24 hours after of tests.

In the second model, the VAS after 24 hours was predicted by HIT-6 (moderate neck disability group) and pain catastrophizing (mild neck disability group), explaining 22% and 14% of the variance, respectively. The VAS (moderate neck disability, β = −0.27; P = 0.06, mild neck disability, β = −0.13; P = 0.41), NDI (moderate neck disability, β = 0.19; P = 0.17, mild neck disability, β = 0.24; P = 0.13), and PCS (moderate neck disability, β = 0.16; P = 0.25), and HIT-6 (mild neck disability, β = −0.054; P = 0.71) were not significant predictors.

In a third model, the pain-free MMO was predicted by NDI for both groups; these models accounted for between 14% and 21% of the variance. The PCS (moderate neck disability, β = 0.20; P = 0.19, mild neck disability, β = 0.13; P = 0.39), the VAS (moderate neck disability, β = −0.34; P = 0.85, mild neck disability, β = −0.26; P = 0.13), and HIT-6 (moderate neck disability, β = −0.24; P = −0.066, mild neck disability, β = 0.20; P = 0.64) were not significant predictors.

## Discussion

The results of this study demonstrate that a protocol of masticatory provocation can induce pain, fatigue, and other masticatory sensory-motor changes in patients with headache attributed to TMD disorders. Our findings are consistent with previous studies that also observed sensory changes induced experimentally by the masticatory provocation test [31,46-48]. The duration of the masticatory provocation test used in our study was similar to other investigations [31,47,49]. However, some studies have used longer or shorter durations for the masticatory test, reporting significant changes in both situations for both patients and healthy subjects [32,46,48,50-52]. It is important to mention that group changes were found in the healthy subjects, but these were smaller than in the other groups, which could be explained by the observation that exercise can induce pain and hyperalgesia [53]. The ability of the masticatory test to provoke pain and fatigue has been proven in several studies, and it is well accepted in both healthy subject and patients with TMD, myofascial pain, or whiplash [31,32,47,51].

We have found a great percentage of subjects in the patient groups with increased intensity of perceived pain and fatigue. However, in the control group with healthy subjects, the increment occurred in a lower percentage of patients, and a significant percentage of patients had no changes. This last point agrees with previous literature that also found a reduction of pain after the test [46,48].

In this regard, our findings show strong positive correlations between fatigue and perceived pain associated with the masticatory provocation test in the three assessed groups. These results may generally explain the observed sensory-motor changes, although they are not sufficient to justify either the between-groups differences or the influence of neck-pain-related disability. Reflections and discussion of these issues are presented in the following section in an effort to achieve a better understanding of the matter.

One of the hypotheses is that neck-pain-related disability has an influence over the masticatory sensory-motor variables and modifies them. The results support this hypothesis, because we observed greater changes in the moderate disability group immediately and 24 hours after the test. In addition, it was hypothesized that the psychosocial factors would have a relationship with the results of the masticatory provocation test and specifically with the pain and fatigue variables. This relationship was proven after observing an association with pain catastrophizing.

### Gender differences

Our data show that gender influences the results of the three groups regarding pain perception and fatigue during the test. Women presented greater perception of pain intensity and masticatory fatigue. These results are consistent with previous studies of experimentally induced pain in patients [47] and healthy subjects [31,52]; however, other investigations have not observed the interaction of gender factors with experimentally induced pain or masticatory fatigue [49,54]. This research has not been designed to identify the physiological or psychological mechanisms, which may explain the differences in the results of men and women. However, it is important to state that there are many studies which present evidence-based results regarding the response that women have to other painful clinical situations, adding to the evidence of experimentally induced pain studies indicating that women have a greater pain sensitivity than men regarding several somatosensory tests [55].

### Influence of neck-pain-related disability over the masticatory sensory-motor activity

We have identified that patients with mild to moderate neck-pain-related disability present greater perception levels of pain and fatigue compared with healthy subjects. It is important to mention that the group with moderate disability presented the greatest changes in the sensorial variables measured during the test, immediately after, and 24 hours later, with the exception of the pain intensity perception after 24 hours, in which no statistically significant differences were found between groups. Although there are many studies that have used a provocative test to induce masticatory pain and fatigue, we have only found one study similar to ours, in which Haggman-Henrikson et al. [47] observed that patients with whiplash-associated disorders presented greater masticatory pain and fatigue induced by the test compared to TMD patients and healthy subjects.

Injuries to the cervical region may alter the masticatory motor control and normal mandibular open-close function [56-58]. The findings of this study may be related to this issue, because the results show that the masticatory provocation test reduces the pain-free MMO at the end of the test in all three groups assessed. These results are similar to previous studies [31,59]. However, the reduction was greater in both patient groups, and it was maintained at 24 hours in only the moderate disability group. Also, the regression analysis showed that neck-pain-related disability is a predictor of the pain-free MMO (after 24 hours) in both groups of patients.

Several authors suggest that nociceptive and motor alterations of the cervical and craniofacial regions could be explained by an anatomical and physiological convergence phenomenon of trigeminal and cervical nociceptive afferents converging in the spinal trigeminal nucleus and the upper cervical segments [60,61], which has been called the trigeminocervical complex (TCC). Evidence from basic studies on animals have demonstrated this phenomenon of convergence [60-66], and there is also evidence of this mechanism in humans [67,68]. The TCC can be sensitized by nociceptive primary afferents from the masseter muscle and TMJ [69-72], and it has also been described that the primary nociceptive afferents from the skin and neck muscles are able to excite neurons at the TCC [65,66,73]. In the sensitization process, efferent outputs that involve connections between motor neurons and neural nociceptive afferents occur, which in turn generate motor responses [74,75]*.* At present, the scientific evidence suggests the existence of cervical and trigeminal motor patterns that act in a coordinated manner in the performance of masticatory activities (chewing) [76-79]. Recent studies also support that the neck muscles are activated during jaw-clenching tasks assessed electromyographically [80-82]. It seems that the activity of the neck muscles is increased with greater demand for masticatory work [83].

As a contributing factor in patients with headache attributed to TMD, the presence of neck pain must be considered, since it can lead to lower values of trigeminal PPTs compared to healthy subjects [84]. We obtained decreased PPTs in the trigeminal and cervical regions, noting that the PPT changes were higher in the patient groups, and that most changes in the cervical PPTs at 24 hours occurred in the group of moderate neck-pain-related disability. Although we believe that there may be a direct relationship of the masticatory sensory-motor changes with cervical pain and disability, we must also consider the possibility that the changes seen in patients could have been mainly influenced by pre-established neuroplastic changes in the central nervous system. Patients with chronic pain may be more susceptible to develop a central sensitization process [85]. Wolf et al. suggest that painful conditions where there is a comorbidity, such as in the sample of patients in this study, can be a determining factor in the pathophysiology of central sensitization [86]. In relation to this, Gaff-Radford proposed that in central sensitization, changes appear in afferent pathways that enable the communication of cervical and orofacial nociceptive neurons in the trigeminal nucleus [87]. In addition, there are many studies on TMD patients that have found peripheral and central mechanisms compatible with a process of central sensitization [88-94].

### Influence of pain catastrophizing over masticatory sensory-motor activity

We have used self-reports of psychological and pain-related variables to identify possible associations with sensory-motor variables. Through linear regression analysis, we have observed that pain catastrophizing and the impact of headache on the quality of life (HIT-6) were associated with the pain perception and fatigue variables 24 hours after performing the masticatory provocation test. Specifically, analysing the pain catastrophizing as a psychological factor resulted in a predictor for fatigue at 24 hours after the test in the moderate neck-pain-related disability group. In the mild disability group, it was a predictor for perceived neck-pain-related disability and fatigue after 24 hours.

Pain catastrophizing is defined as a cognitive factor that implies a mental negative perception or exaggeration of the perceived threat of either a real or anticipated pain experience [95,96]. It has been described that in patients with TMD, catastrophizing contributes to the chronification of pain and disability [22]. It has also been associated with a greater use of health system services, greater clinical findings at assessment associated with a negative mood [24,25], and alterations of the functional mandibular status [1]. Regarding the perceived fatigue and pain catastrophizing, we did not find any clinical or experimental trials that have examined their association in patients with craniofacial pain and TMD, but we found one study researching the relationship of pain catastrophizing with masticatory kinematic variables (i.e., amplitude, velocity, frequency cycle), which were measured with a procedure using very short exposure times (15 seconds of chewing) [16]. In this study, no associations of the kinematic variables measured with respect to catastrophism were observed; however, we must take into account that the purpose of the previous study was not to induce pain or fatigue to observe the response, as we did in this research. It is important to note that a recent systematic review concluded that there is an association between catastrophizing and fatigue, and that the former influences the latter proportionately. These results were observed in various clinical populations [97]. This has also been demonstrated in other musculoskeletal disorders where pain catastrophizing is associated with motor disturbances, such as decreased function, performance of activities of daily living, and limitation of exercise capacity [98-100].

The relationship between psychological factors, motor activity, and pain seems to be present in various cases of musculoskeletal pain, but the explanation for this is complex and limited so far. Peck et al. [101] and Murray and Peck [102] proposed a possible explanation for this and created a new Integrated Pain Adaptation Model (IPAM). This model basically explains that the influence of pain on motor activity depends on the interaction of multidimensional characteristics (biological and psychosocial) of pain with the sensory-motor system of an individual, which results in a new motor recruitment strategy to minimize pain. However, this motor response may be associated with the appearance of other pain or worsening of the existing pain [101,102]. This model is based on the multidimensional features (sensory discriminative, affective-emotional, cognitive) of the pain experience and how they affect the sensory-motor system through the peripheral and central connections that this system has with the autonomous nervous system, limbic system, and other higher centres [101,103].

#### Clinical and scientific implications

The results showed that neck pain and disability can influence sensory and motor variables of the masticatory system. These findings lead us to reflect on the importance of including a clinically specific assessment of the cervical region in the diagnostic protocols for TMD and headache attributed to TMD. It is noteworthy that the most commonly used diagnostic and classification methods for patients with TMD do not include a specific assessment of neck pain and disability [4,28,104]. A diagnostic criterion observed recently in patients with headache attributed to TMD is that mandibular movement, function, or parafunction modify headache in the temporal region [5]. We have observed an association between neck-pain-related disability and pain-free MMO, and we have also found that patients with greater neck disability have increased fatigue and pain induced by the masticatory test. These findings lead us to assume that the cervical region may have an important role for this type of headache, but this has to be confirmed in future research, as these data can be extrapolated only to patients with this type of headache who also have neck disability.

From the perspective of treatment, we propose an approach to reduce cervical pain and disability as part of the overall therapeutic strategy, which could be beneficial to reduce the negative sensory symptoms and improve masticatory motor control. We believe that this approach should be investigated in future studies, but it must be taken into account that we have recent evidence that therapeutic exercise and manual therapy to the cervical region produce positive effects on pain modulation in trigeminal areas and improving pain-free MMO [30,105].

This study and other longitudinal or transversal studies has shown the influence of psychosocial factors on patients with TMD [20,106,107]. Specifically, our results show an association between catastrophizing and perceived fatigue induced by the masticatory activity. This finding shows the interaction between sensory-type variables with psychological variables, which should be considered a crucial issue when assessing or designing therapeutic interventions. In patients with chronic pain, it is essential to recognize psychosocial factors that may be perceived as obstacles to recovery [108]. Achieving a reduction of pain catastrophizing is the best predictor of successful rehabilitation in pain conditions [109].

The integration of a biopsychosocial perspective in clinical reasoning and decision making could be a key point in the management of pain and motor rehabilitation of patients with headache attributed to TMD. It has been shown that cognitive behavioural therapy reduces pain intensity and depressive symptoms, improves chewing function [110], and reduces pain catastrophizing in patients with chronic TMD [111]. Furthermore, it has been found that it causes neuroplastic adaptive changes associated with decreased pain catastrophism in cases of chronic pain [112]. Prescribing therapeutic exercise may be a good alternative to take into consideration. It has been observed that exercise causes a reduction of catastrophizing and depressive symptoms, and these results were similar to cognitive behavioural therapy in patients with chronic lower back pain [113].

### Limitations

Although the sample size was calculated to have adequate power and further losses were less than 20%, the results were not compared with a group with headache attributed to TMD but without the presence of neck pain and disability. Extrapolating the results to a clinical population would require similar future studies to be implemented using patient sample protocols with and without neck pain and disability. Another limitation to consider is that pain catastrophizing was only psychological variable assessed. It would be interesting to investigate the association of other variables such as anxiety, depression, kinesiophobia, and self-efficacy with masticatory sensory-motor variables.

More variables are needed to better quantify the sensory-motor system activity, such as electromyography, jaw-stretch reflex, and temporal summation. The only motor variable measured was pain-free MMO, but other kinematic variables should be taken into consideration in future research, as they may provide more information about the motor system. Moreover, we believe that measuring motor variables of the cervical region could also be useful to analyse possible correlations with masticatory variables.

Another limitation is related to the of TMD-related disability measurements, which could influence the performance of the provocation test. At the time that the study was performed, there was no TMD disability index in Spanish, but one has been developed [1].

## Conclusion

The results of this study suggest that neck pain and disability have an influence on the sensory-motor variables evaluated in patients with headache attributed to TMD. In particular, patients with moderate neck disability showed greater changes immediately and 24 hours after the masticatory provocation test. Our data provide new evidence about the possible neurophysiologic mechanisms of interaction between the craniocervical region and the craniomandibular region. Regarding pain catastrophizing, an association with perceived masticatory fatigue in both patient groups was observed. These findings support the need to recognize the interaction between sensory-motor and psychological aspects of headache attributed to TMD rather than assessing them isolation.
